# Supplementary material for: A Quantitative Modular Modeling Approach Reveals the Effects of Different A20 Feedback Implementations for the NF-kB Signaling Dynamics
Source: Front Physiol. 2020 Jul 28;11:896. doi: 10.3389/fphys.2020.00896 (PMC7402004; doi:10.3389/fphys.2020.00896)
Supplement: Supplementary file 1 [file Data_Sheet_1.pdf]

Supplementary Information

A quantitative modular modeling approach reveals the effects of different A20 feedback implementations for the NF-kB signaling dynamics

Contents

List of supplementary Tables 1

List of supplementary Figures 2

ODE of mathematical models 3

Parameter estimation of the upstream modules 5

Dynamics of all components in models A-C 8

Sensitivity analyses of models A-C 10

NF-κB dynamics for low A20 feedback strengths and high stimulation strengths in model A 14

Prediction of the experimental outcome for different TNFα stimulations 14

EMSA replicate experiments 16

Supplementary references 17

List of supplementary Tables

Supplementary Table S1. The parameters of the core model and the estimated parameters of the three upstream modules. 7

List of supplementary Figures

Supplementary Figure S1. The dynamics of NF-κB for models A, B and C with estimated parameters for the upstream modules assuming different RC3H1 expression levels. 8
Supplementary Figure S2. The dynamics of all components for the model A, model B and model C. 8
Supplementary Figure S3. Simulations of A20 KO of model A. 9
Supplementary Figure S4. Simulations of A20 KO of model B. 9
Supplementary Figure S5. Simulations of A20 KO of model C. 10
Supplementary Figure S6. Sensitivity analysis of the three models regarding the maximal NF-κB concentration. 11
Supplementary Figure S7. Sensitivity analysis of the three models regarding the time of the maximal NF-κB concentration. 12
Supplementary Figure S8. Sensitivity analysis of the three models regarding the response time of NF-κB concentration. 13
Supplementary Figure S9. Simulated NF-κB dynamics and corresponding response time for stimulation strength of 100 and A20 feedback strengths of 1 (solid line), 0.1 (dotted line) and 0.01 (dashed line). 14
Supplementary Figure S10. Simulated NF-κB dynamics upon stimulation with 10 ng/ml (solid line), 25 ng/ml (dotted line) and 100 ng/ml TNFα (dashed line) for A20 feedback strength set to 10 (first row), 1 (second row) and 0.1 (third row) for model A (first column), model B (second column) and model C (third column). Stimulus of 1 au equals a TNFα concentration of 10 ng/ml. 15
Supplementary Figure S11. Simulated NF-κB dynamics upon stimulation with 10 ng/ml (solid line), 25 ng/ml (dotted line) and 100 ng/ml TNFα (dashed line) for the models published by Murakawa et al. (2015) (first column), Ashall et al. (2009) (second column) and Lipniacki et al. (2004) (third column). Stimulus of 1 au equals a TNFα concentration of 10 ng/ml. 15
Supplementary Figure S12. Replicate EMSA experiments measuring NF-κB DNA-binding activity upon stimulation with different TNFα concentrations. A, B: Exemplary EMSA experiments measuring NF-κB DNA-binding activity over a time course of 120 min in HeLa cells upon stimulation with 10 ng/ml, 25 ng/ml and 100 ng/ml TNFα. The histograms show the respective quantifications of the EMSA experiments. The mean value of the relative intensity at t=0 is set to 1 and used as a normalization for all other values. 16

ODE of mathematical models

Core module:

$$\frac{d}{dt}A20_{mRNA}(t) = v_{10} - v_6$$

$$\frac{d}{dt}A20(t) = v_8 - v_3$$

$$\frac{d}{dt}IkBa(t) = v_9 - v_4 - v_1$$

$$\frac{d}{dt}IkBa_{mRNA}(t) = v_{11} - v_7$$

$$\frac{d}{dt}NFkB|IkBa(t) = v_1 - v_5$$

$$\frac{d}{dt}NFkB(t) = -v_1 + v_5$$

The conservation relation of NFκB is implemented by setting the initial concentrations of the complex NFκB|IκBα to 0 au and the initial concentration of NFκB to $NF\kappa B_{total}$:

$$NFkB_{total} = NFkB|IkBa(t) + NFkB(t) = k_{12}$$

Upstream module A:

$$\frac{d}{dt}IKK_{active}(t) = v_{14} - v_{15} + v_{13}$$

Upstream module B:

$$\frac{d}{dt}IKK_{neutral}(t) = v_{18} - v_{16}$$

$$\frac{d}{dt}IKK_{active}(t) = v_{16} - v_{17}$$

$$\frac{d}{dt}IKK_{inactive}(t) = v_{17} - v_{18}$$

The conservation relation of IKK is implemented by setting the initial concentrations of IKK_{active} and $IKK_{inactive}$ to 0 au and the initial concentration of $IKK_{neutral}$ to IKK_{total}:

$$IKK_{total} = IKK_{neutral}(t) + IKK_{active}(t) + IKK_{inactive}(t) = k_{20}$$

Upstream module C:

$$\frac{d}{dt}IKK_{neutral}(t) = v_{24} - v_{25} - v_{21}$$

$$\frac{d}{dt}IKK_{active}(t) = v_{21} - v_{23} - v_{22} - v_{26}$$

$$\frac{d}{dt}IKK_{inactive}(t) = v_{23} + v_{22} - v_{27}$$

Reactions:

$$v_1 = k_1 * IkBa(t) * NFkB(t) - k_2 * NFkB|IkBa(t)$$

$$v_3 = k_3 * A20(t)$$

$$v_4 = k_4 * IkBa(t)$$

$$v_5 = k_5 * NFkB|IkBa(t) * IKK_{active}(t)$$

$$v_6 = k_6 * A20_{mRNA}(t)$$

$$v_7 = k_7 * IkBa_{mRNA}(t)$$

$$v_8 = k_8 * A20_{mRNA}(t)$$

$$v_9 = k_9 * IkBa_{mRNA}(t)$$

$$v_{10} = k_{10} * NFkB(t)$$

$$v_{11} = k_{11} * NFkB(t)$$

$$v_{13} = stimulus * k_{13} * e^{-A20(t)}$$

$$v_{14} = \frac{k_{14}}{k_{14} - A20(t)}$$

$$v_{15} = k_{15} * IKK_{active}(t)$$

$$v_{16} = stimulus * k_{16} * IKK_{neutral}(t)$$

$$v_{17} = k_{17} * IKK_{active}(t)$$

$$v_{18} = k_{18} * IKK_{inactive}(t) * \frac{k_{19}}{k_{19} + A20(t) * stimulus}$$

$$v_{21} = stimulus * k_{21} * IKK_{neutral}(t)$$

$$v_{22} = stimulus * k_{22} * IKK_{active}(t) * A20(t)$$

$$v_{23} = k_{23} * IKK_{active}(t)$$

$$v_{24} = k_{24}$$

$$v_{25} = k_{25} * IKK_{neutral}(t)$$

$$v_{26} = k_{26} * IKK_{active}(t)$$

$$v_{27} = k_{27} * IKK_{inactive}(t)$$

Stimulation:

$$stimulus(t) = \begin{cases} 0 & if \ \ t \leq 0 \\ 1 & if \ \ t > 0 \end{cases}$$

Parameter estimation of the upstream modules

We choose the dynamics of the model by Murakawa *et al.* (2015) as our reference dynamics. The latter model was generated to reproduce the dynamics of three different experimental scenarios: wild type cells and cells where the RNA-binding protein RC3H1 is either overexpressed or knocked-down. RC3H1 was identified to influence the degradation of A20 mRNA and IκBα mRNA. For all three scenarios, wild type, RC3H1 overexpression and RC3H1 knock-down, the dynamics of active IKK and A20 are simulated with the original model by Murakawa *et al.* (2015). The simulated dynamics for the two components are used as input data for the estimation of the parameters of the upstream modules of our three models A, B and C. Based on the input data, the upstream module parameters are estimated. Thus, we applied the D2D toolbox for Matlab (R2013b, The MathWorks Inc., Natick, MA) using a deterministic

optimization algorithm with multi-start parameter sampling (Raue et al., 2013). As deterministic optimizations may converge to a local rather than a global optimum, the multi-start approach is used to compensate for this limitation (Raue et al., 2013).

For the parameter sampling, we used Latin hypercube sampling as proposed by (Raue et al., 2013). It is a type of stratified sampling, where the parameter space is divided into equi-probable regions and samples are drawn from these regions without replacement, generating a global selection of starting points. This ensures that each optimization run starts in a different region of the high-dimensional parameter space.

In the D2D toolbox the negative logarithm of the maximum likelihood estimator is used to calibrate the dynamical model, where the observables y are compared to the simulated data $\hat{y}$ given the model parameter θ:

$$-2\ln(L(\hat{y}|\theta)) = \sum_{k=1}^{n}\sum_{i=1}^{d_k}(\ln(2\pi\sigma_{ki}^2) + \frac{(\hat{y}_{ki} - y_k(t_i,\theta))^2}{\sigma_{ki}^2}$$

d_k denotes the number of simulated data $\hat{y}$ for each observable $k = 1, \ldots, n$ measured at time points t_i with $i = 1, \ldots, d_k$. The variance components of the noise of each data point are denoted by $\sigma_{d_k}^2$. The in-build function *lsqnonlin* of Matlab (R2013b, The MathWorks Inc., Natick, MA) was used to minimize this function with the constraints of non-negative observables y.

We sampled 10000 start parameter sets and at least 20% of the sampled start parameter sets converged to the optimal parameter set during optimization, suggesting that we found a global optimum. The model parameters are shown in **Supplementary Table S1**.

Parameter	Value	unit	module	Description
k_1	9727.71	$au^{-1}min^{-1}$	core	association of IκBα and NF-κB
k_2	39.28	min^{-1}	core	dissociation of NFκB|IκBα
k_3	$8.59*10^{-1}$	min^{-1}	core	A20 protein degradation
k_4	$5.5*10^{-3}$	min^{-1}	core	IκBα protein degradation
k_5	$6.3*10^{-3}$	$au^{-1}min^{-1}$	core	IKK-induced IκBα protein degradation
k_6	$3.0*10^{-4}$	$au^{-1}min^{-1}$	core	A20 mRNA degradation
k_7	$3.86*10^{-4}$	$au^{-1}min^{-1}$	core	IκBα mRNA degradation
k_8	$1.0*10^{-2}$	min^{-1}	core	A20 protein synthesis
k_9	$1.3*10^{-1}$	min^{-1}	core	IκBα protein synthesis
k_{10}	$9.88*10^{-1}$	min^{-1}	core	A20 mRNA synthesis
k_{11}	$1.59*10^{-3}$	min^{-1}	core	IκBα mRNA synthesis
k_{12}	1.45	au	core	total NF-κB concentration
k_{13}	12.66	min^{-1}	A	TNFα-induced IKK activation
k_{14}	$1.18*10^{-3}$	au	A	basal IKK activation
k_{15}	$2.23*10^{-1}$	min^{-1}	A	IKK inactivation
k_{16}	$1.42*10^{-1}$	min^{-1}	B	TNFα-induced IKK activation
k_{17}	$2.7*10^{-2}$	min^{-1}	B	basal IKK inactivation
k_{18}	1000	$au^{-1}min^{-1}$	B	recycling of inactive IKK
k_{19}	$6.44*10^{-6}$	au	B	IKK inhibition by A20
k_{20}	51.65	au	B	total IKK concentration
k_{21}	$1.18*10^{-2}$	min^{-1}	C	TNFα-induced activation of IKK
k_{22}	$6.82*10^{-2}$	$au^{-1}min^{-1}$	C	TNFα-induced IKK inactivation
k_{23}	$1.6*10^{-1}$	min^{-1}	C	basal IKK inactivation
k_{24}	1.33	au	C	synthesis of neutral IKK
k_{25}	$2.05*10^{-3}$	min^{-1}	C	degradation of neutral IKK
k_{26}	$5.72*10^{-4}$	min^{-1}	C	degradation of active IKK
k_{27}	$1.9*10^{-9}$	min^{-1}	C	degradation of inactive IKK

Supplementary Table S1. The parameters of the core model and the estimated parameters of the three upstream modules.

All three refitted models reproduce the experimental observations for the different expression levels of the RNA-binding protein RC3H1 utilized in Murakawa *et al.* (2015) (**Supplementary Figure S1**).

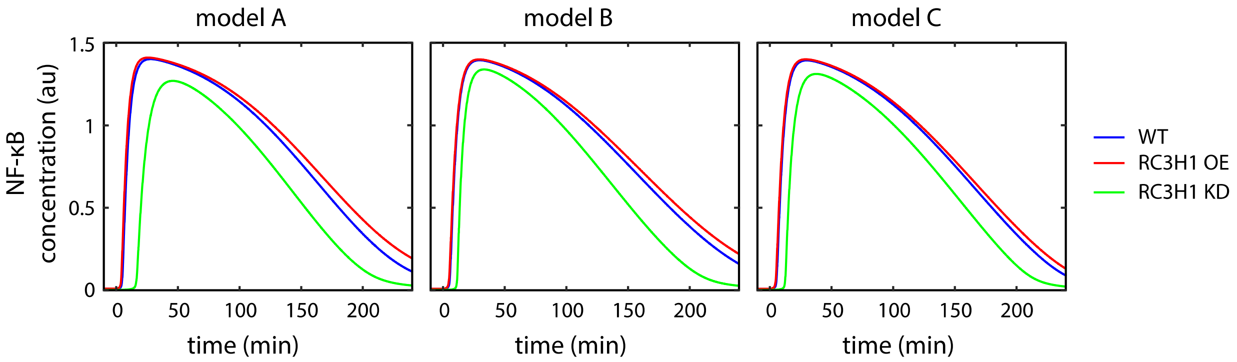

Supplementary Figure S1. The dynamics of NF-κB for models A, B and C with estimated parameters for the upstream modules assuming different RC3H1 expression levels.

Dynamics of all components in models A-C

A comparison of the dynamics of all components of models A-C is shown in **Supplementary Figure S2**. The components of the core module (NF-κB|IκBα, NF-κB, IκBα, A20, A20 mRNA, IκBα mRNA) are nearly identical in all three models. In addition, the dynamics of active IKK shows also similarities for all three models. Differences can be observed for the dynamics of neutral IKK and inactive IKK in the model B and C. In model A, neutral IKK and inactive IKK do not exist.

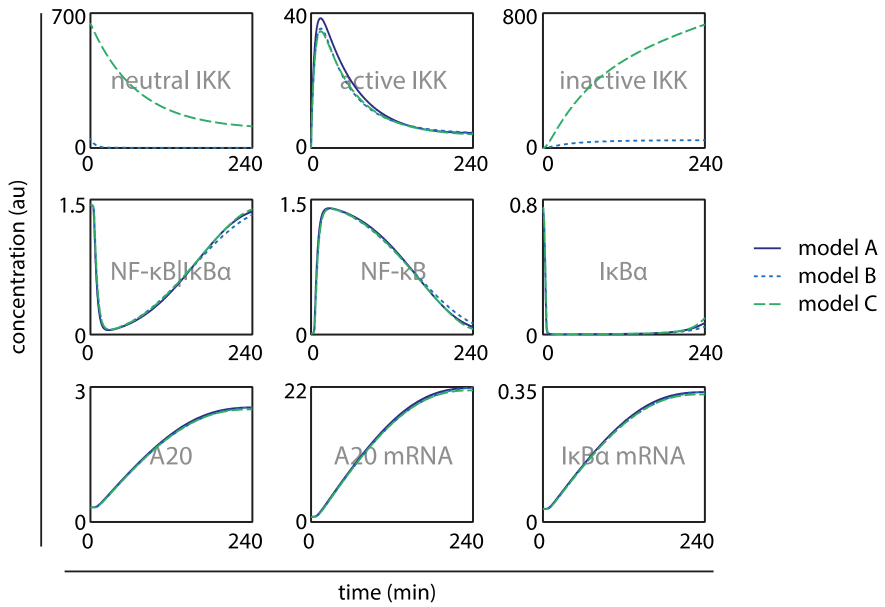

Supplementary Figure S2. The dynamics of all components for the model A, model B and model C.

For a first validation of the models with refitted parameters, we qualitatively compare the models to experimental data published by (Lee et al., 2000), where concentrations of key components were measured in wild type cells and A20 knock-out cells. Experiments showed that in A20 knock-out cells the NF-κB activation and the IκBα degradation is prolonged compared to wild type cells. Simulations of all three models qualitatively reproduce the experimentally observed dynamical changes (**Supplementary Figure S3** – **Supplementary Figure S5**). The activity of IKK and NF-κB is prolonged and the IκBα mRNA level is increased, whereas the IκBα protein level is decreased due to the increased levels of active IKK. Thus, the combination of the different modules and newly estimated parameters are able to reproduce the general, inhibitory effect of A20 on IKK.

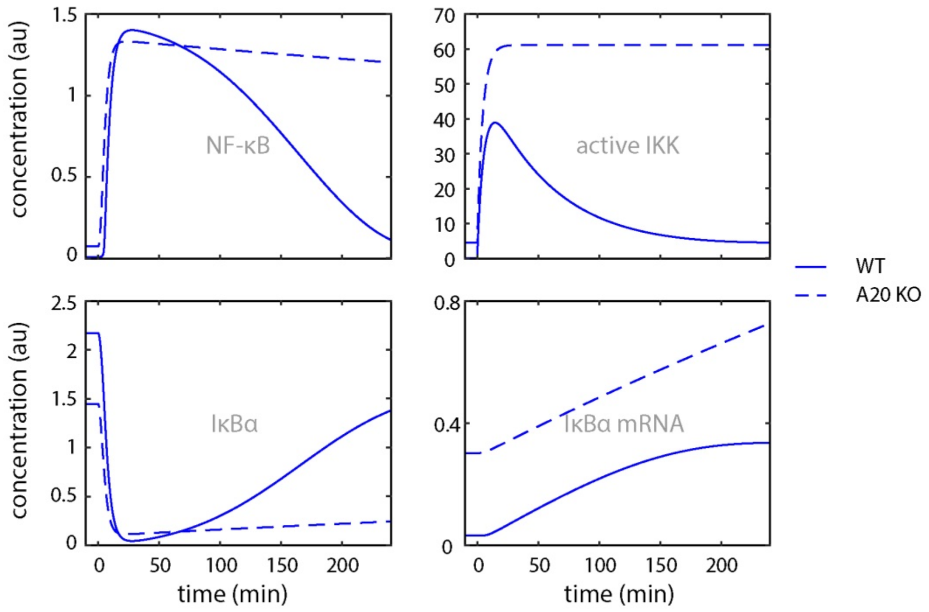

Supplementary Figure S3. Simulations of A20 KO of model A.

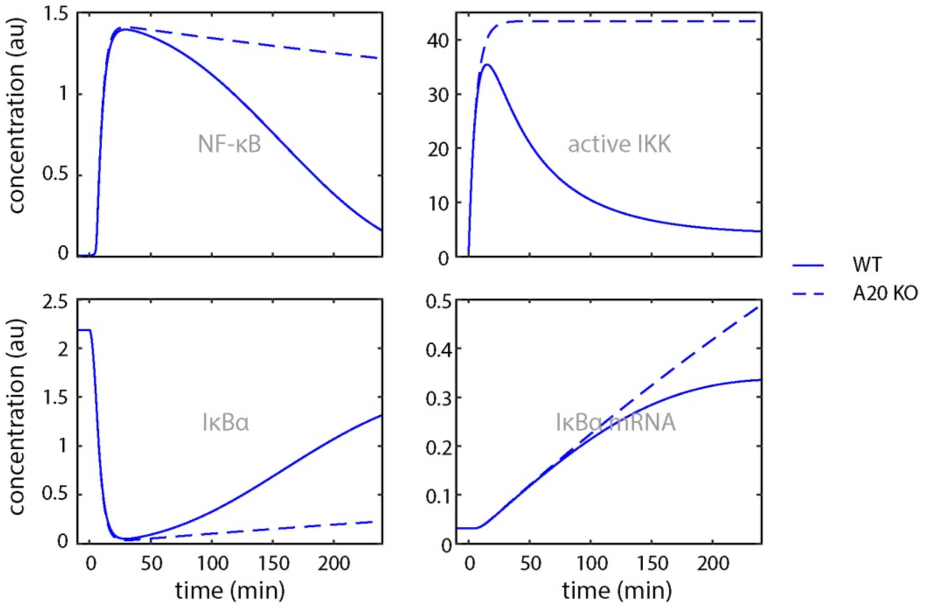

Supplementary Figure S4. Simulations of A20 KO of model B.

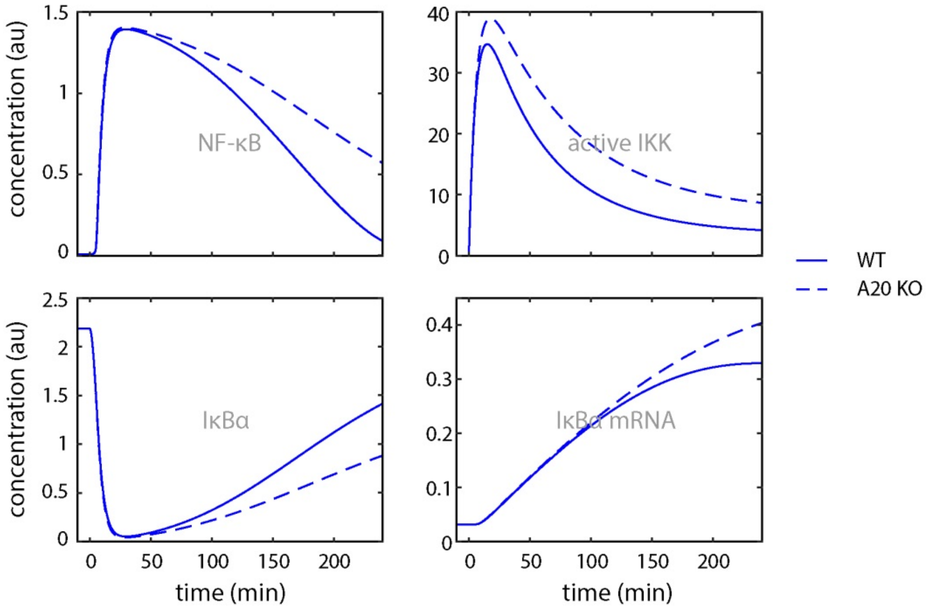

Supplementary Figure S5. Simulations of A20 KO of model C.

Sensitivity analyses of models A-C

Supplementary Figure S6 – **Supplementary Figure S8** show sensitivity analyses for the three measures: maximal concentration, time of maximal concentration and response time of NF-κB. The sensitivity analyses show the influence of each model parameter on the accordant measure. The sensitivity coefficients for each of the three measures for the A20 mRNA synthesis and the IκBα mRNA synthesis are similar to the A20 protein synthesis and the IκBα protein synthesis, respectively. In conclusion, varying the mRNA synthesis has a similar influence on the measures as varying the protein synthesis. Thus, changes in the A20 or IκBα feedback strength is represented by varying the A20 or IκBα mRNA synthesis, respectively.

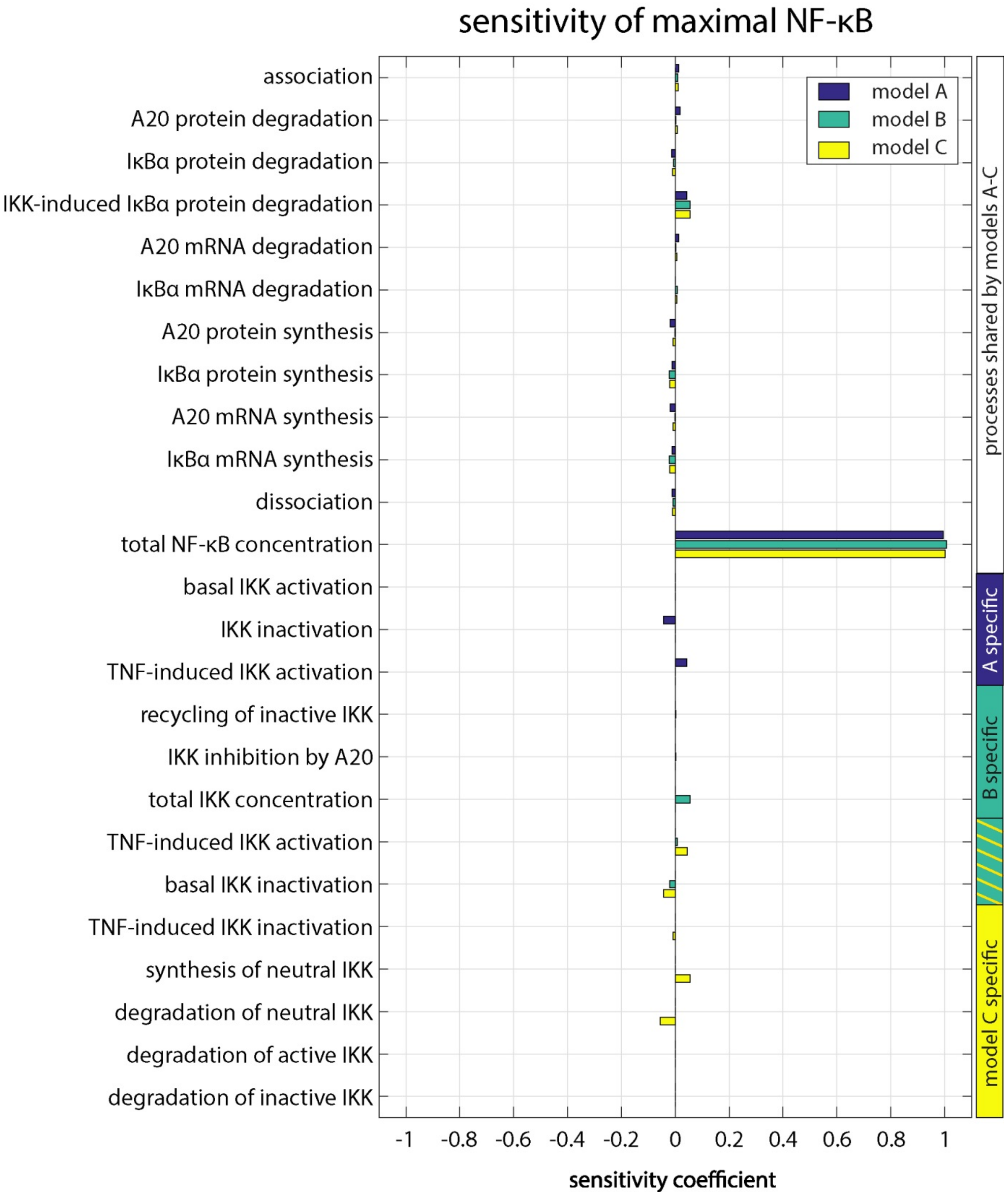

Supplementary Figure S6. Sensitivity analysis of the three models regarding the maximal NF-κB concentration.

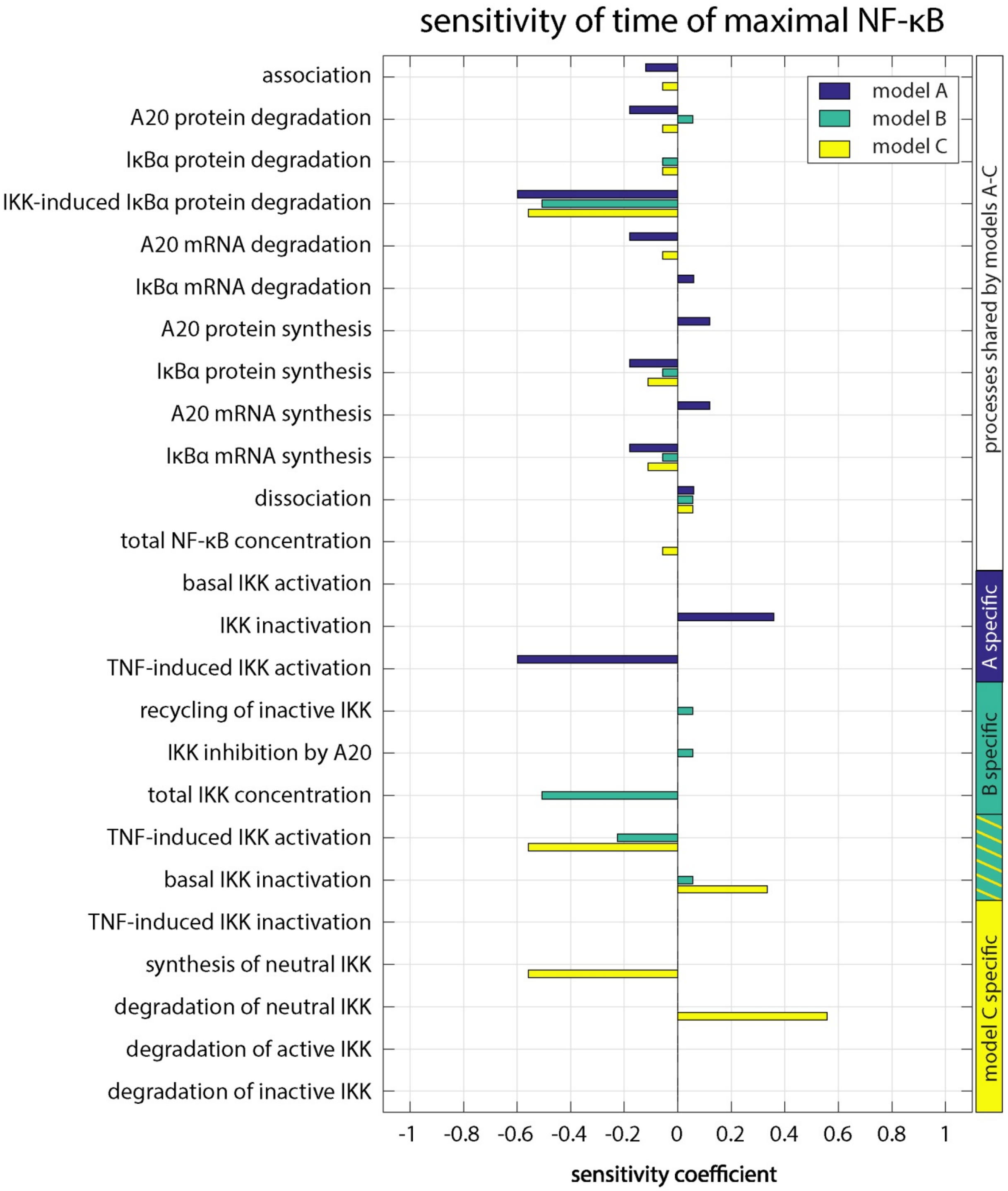

Supplementary Figure S7. Sensitivity analysis of the three models regarding the time of the maximal NF-κB concentration.

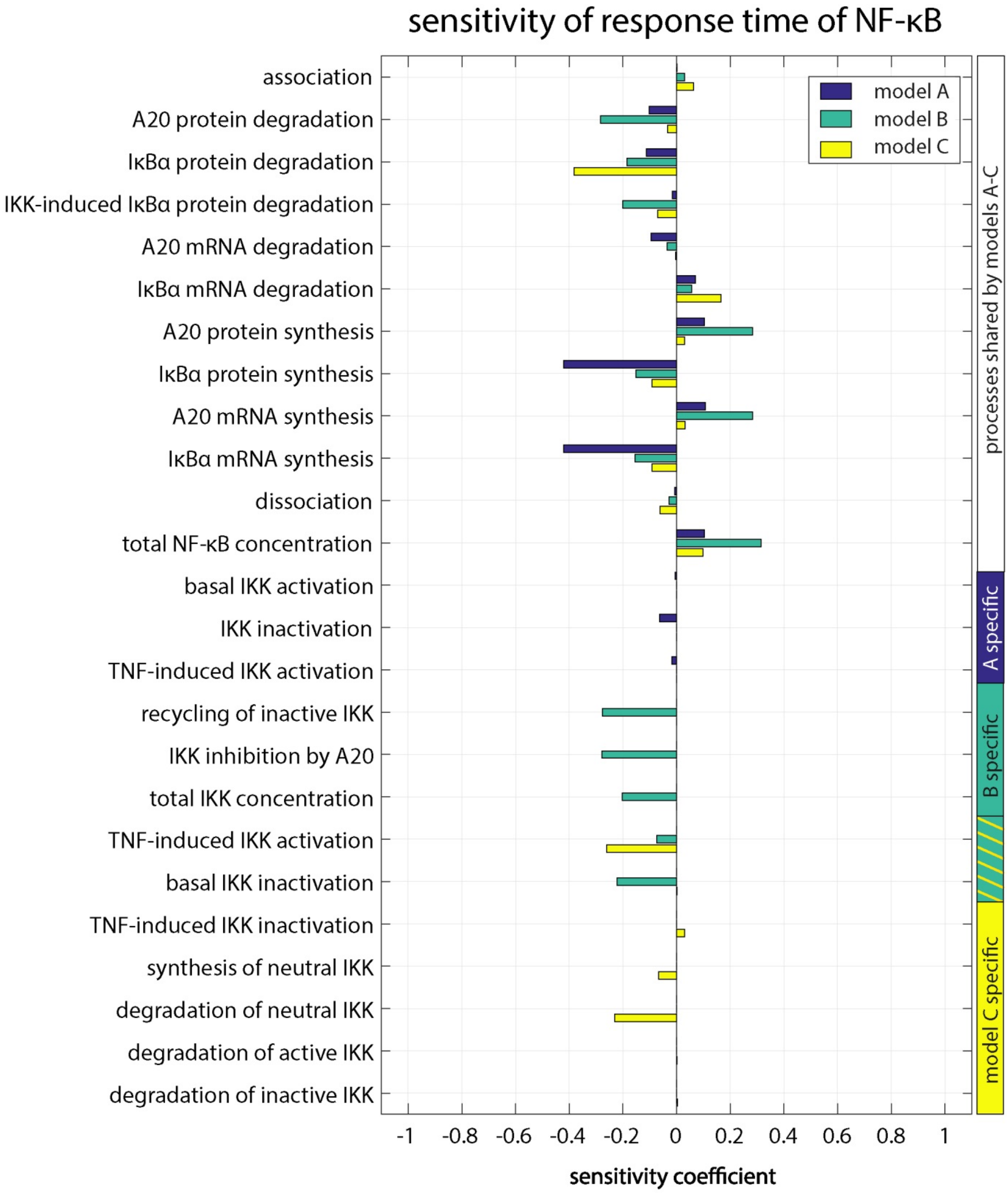

Supplementary Figure S8. Sensitivity analysis of the three models regarding the response time of NF-κB concentration.

NF-κB dynamics for low A20 feedback strengths and high stimulation strengths in model A

Supplementary Figure S9 shows three exemplary NF-κB dynamics and their corresponding response times for a stimulation strength of 100 and A20 feedback strengths of 1, 0.1 and 0.01 to illustrate the distinct increase in the response time for low A20 feedback strengths and high stimulation strengths in model A (Figure 5 – first column, third row).

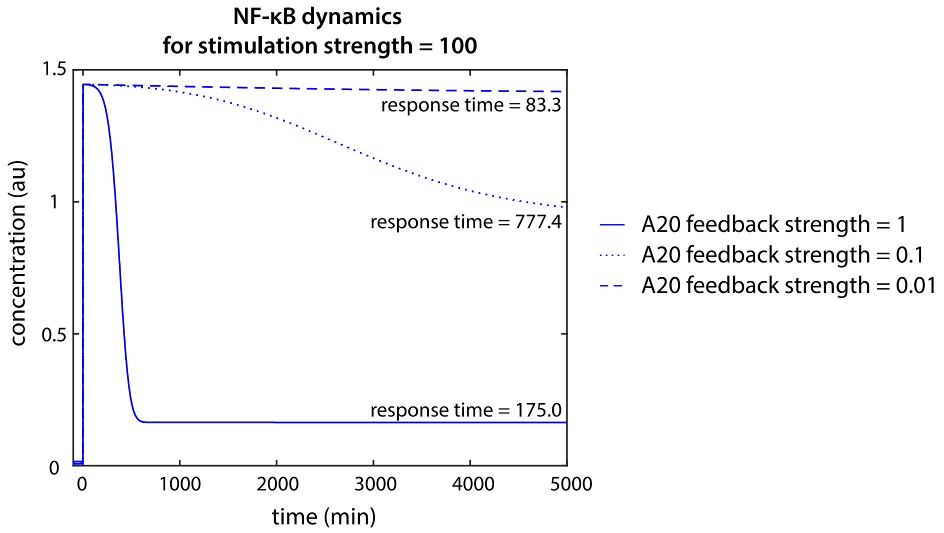

Supplementary Figure S9. Simulated NF-κB dynamics and corresponding response time for stimulation strength of 100 and A20 feedback strengths of 1 (solid line), 0.1 (dotted line) and 0.01 (dashed line).

Prediction of the experimental outcome for different TNFα stimulations

In **Supplementary Figure S10** the dynamics of NF-κB of model A, B and C are shown for the three different strengths of stimulation in the case of high, intermediate and low A20 feedback strength. Model A for intermediate and low A20 feedback strength is in qualitative agreement with the experimental results showing a delayed and reduced decrease in NF-κB concentration for higher concentration of TNFα compared to low TNFα concentrations.

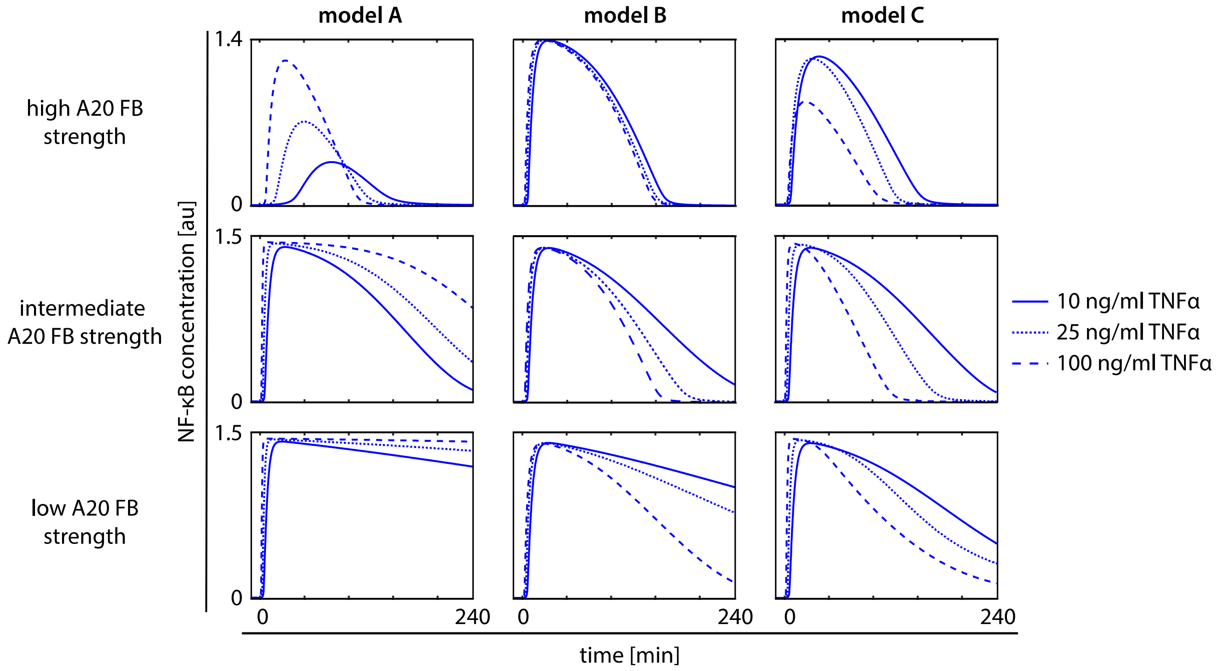

Supplementary Figure S10. Simulated NF-κB dynamics upon stimulation with 10 ng/ml (solid line), 25 ng/ml (dotted line) and 100 ng/ml TNFα (dashed line) for A20 feedback strength set to 10 (first row), 1 (second row) and 0.1 (third row) for model A (first column), model B (second column) and model C (third column). Stimulus of 1 au equals a TNFα concentration of 10 ng/ml.

In **Supplementary Figure S11** the dynamics of NF-κB is shown for the three different strengths of stimulation for the original models published by Murakawa *et al.* (2015), Ashall *et al.* (2009) and Lipniacki *et al.* (2004).

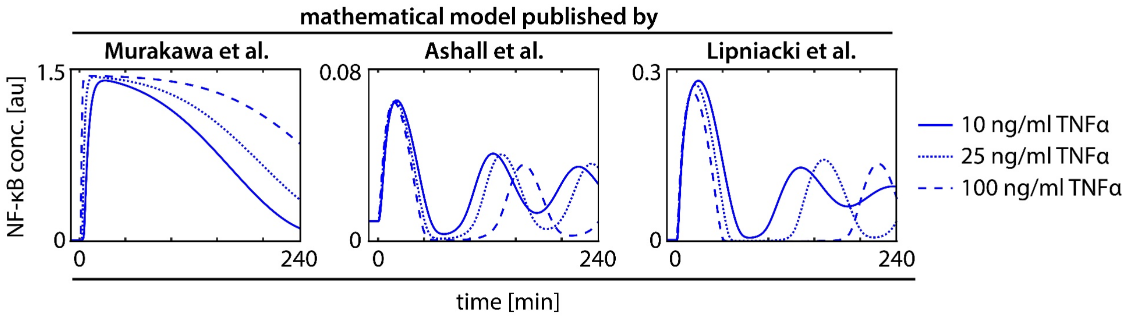

Supplementary Figure S11. Simulated NF-κB dynamics upon stimulation with 10 ng/ml (solid line), 25 ng/ml (dotted line) and 100 ng/ml TNFα (dashed line) for the models published by Murakawa *et al.* (2015) (first column), Ashall *et al.* (2009) (second column) and Lipniacki *et al.* (2004) (third column). Stimulus of 1 au equals a TNFα concentration of 10 ng/ml.

EMSA replicate experiments

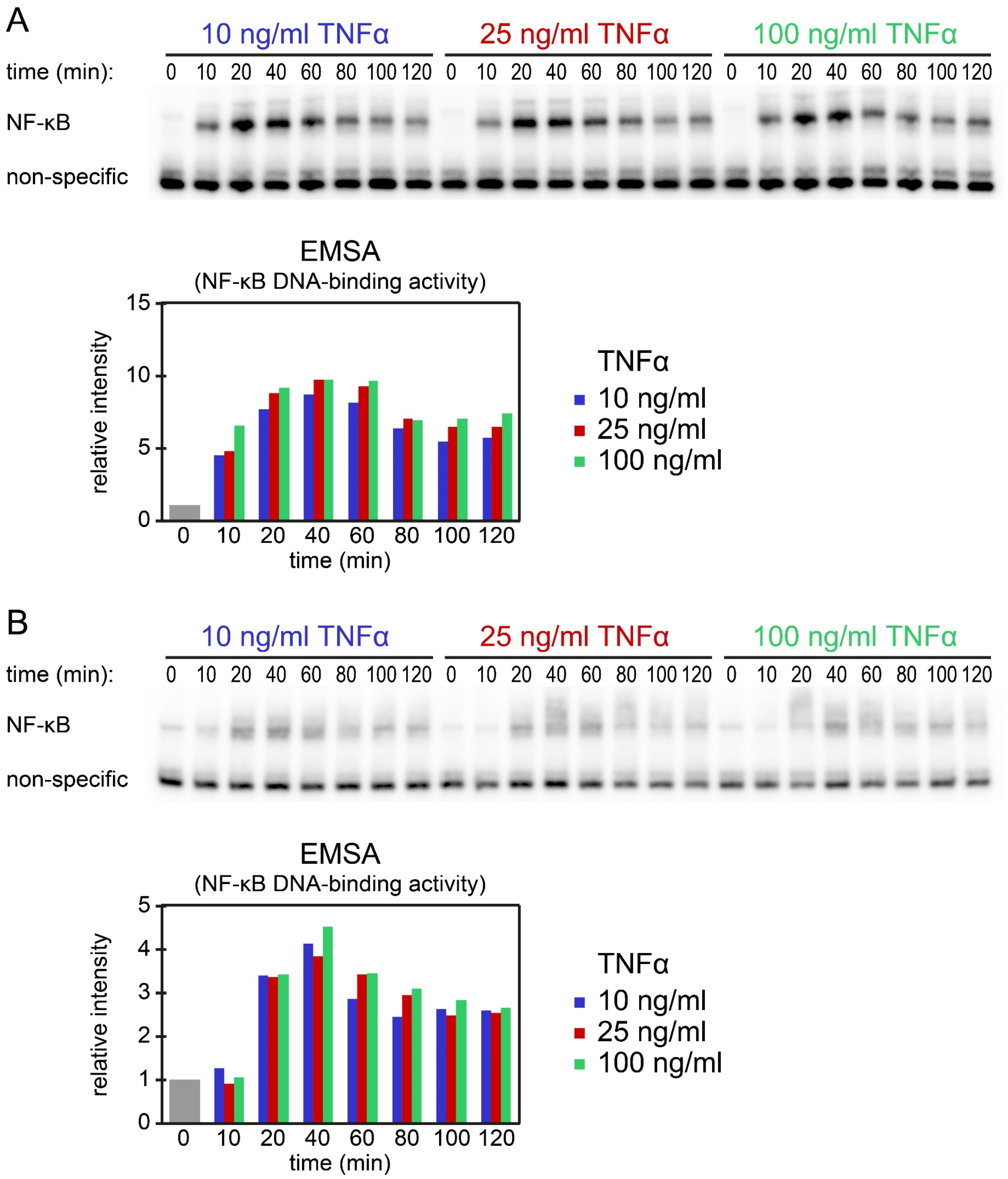

Supplementary Figure S12. Replicate EMSA experiments measuring NF-κB DNA-binding activity upon stimulation with different TNFα concentrations. A, B: Exemplary EMSA experiments measuring NF-κB DNA-binding activity over a time course of 120 min in HeLa cells upon stimulation with 10 ng/ml, 25 ng/ml and 100 ng/ml TNFα. The histograms show the respective quantifications of the EMSA experiments. The mean value of the relative intensity at t=0 is set to 1 and used as a normalization for all other values.

Supplementary references

Ashall, L., Horton, C. A., Nelson, D. E., Paszek, P., Harper, C. V., Sillitoe, K., Ryan, S., Spiller, D. G., Unitt, J. F., Broomhead, D. S., Kell, D. B., Rand, D. A., See, V. and White, M. R. (2009) 'Pulsatile stimulation determines timing and specificity of NF-kappaB-dependent transcription', *Science,* 324(5924), pp. 242-6.

Lee, E. G., Boone, D. L., Chai, S., Libby, S. L., Chien, M., Lodolce, J. P. and Ma, A. (2000) 'Failure to regulate TNF-induced NF-kappaB and cell death responses in A20-deficient mice', *Science,* 289(5488), pp. 2350-4.

Lipniacki, T., Paszek, P., Brasier, A. R., Luxon, B. and Kimmel, M. (2004) 'Mathematical model of NF-kappaB regulatory module', *J Theor Biol,* 228(2), pp. 195-215.

Murakawa, Y., Hinz, M., Mothes, J., Schuetz, A., Uhl, M., Wyler, E., Yasuda, T., Mastrobuoni, G., Friedel, C. C., Dolken, L., Kempa, S., Schmidt-Supprian, M., Bluthgen, N., Backofen, R., Heinemann, U., Wolf, J., Scheidereit, C. and Landthaler, M. (2015) 'RC3H1 post-transcriptionally regulates A20 mRNA and modulates the activity of the IKK/NF-kappaB pathway', *Nat Commun,* 6, pp. 7367.

Raue, A., Schilling, M., Bachmann, J., Matteson, A., Schelker, M., Kaschek, D., Hug, S., Kreutz, C., Harms, B. D., Theis, F. J., Klingmuller, U. and Timmer, J. (2013) 'Lessons learned from quantitative dynamical modeling in systems biology', *PloS one,* 8(9), pp. e74335.
